# Structure of human aspartyl aminopeptidase complexed with substrate analogue: insight into catalytic mechanism, substrate specificity and M18 peptidase family

**DOI:** 10.1186/1472-6807-12-14

**Published:** 2012-06-21

**Authors:** Apirat Chaikuad, Ewa S Pilka, Antonio De Riso, Frank von Delft, Kathryn L Kavanagh, Catherine Vénien-Bryan, Udo Oppermann, Wyatt W Yue

**Affiliations:** 1 Structural Genomics Consortium, Old Road Research Campus Building, Oxford OX3 7DQ, UK; 2 Laboratory of Molecular Biophysics, Department of Biochemistry, Oxford, OX1 3QU, UK; 3 Botnar Research Centre, Oxford Biomedical Research Unit, Oxford, OX3 7LD, UK

**Keywords:** Aspartyl aminopeptidase, Dodecameric tetrahedron, M18 peptidase, Metalloprotease, Domain swapping

## Abstract

**Backround:**

Aspartyl aminopeptidase (DNPEP), with specificity towards an acidic amino acid at the N-terminus, is the only mammalian member among the poorly understood M18 peptidases. DNPEP has implicated roles in protein and peptide metabolism, as well as the renin-angiotensin system in blood pressure regulation. Despite previous enzyme and substrate characterization, structural details of DNPEP regarding ligand recognition and catalytic mechanism remain to be delineated.

**Results:**

The crystal structure of human DNPEP complexed with zinc and a substrate analogue aspartate-β-hydroxamate reveals a dodecameric machinery built by domain-swapped dimers, in agreement with electron microscopy data. A structural comparison with bacterial homologues identifies unifying catalytic features among the poorly understood M18 enzymes. The bound ligands in the active site also reveal the coordination mode of the binuclear zinc centre and a substrate specificity pocket for acidic amino acids.

**Conclusions:**

The DNPEP structure provides a molecular framework to understand its catalysis that is mediated by active site loop swapping, a mechanism likely adopted in other M18 and M42 metallopeptidases that form dodecameric complexes as a self-compartmentalization strategy. Small differences in the substrate binding pocket such as shape and positive charges, the latter conferred by a basic lysine residue, further provide the key to distinguishing substrate preference. Together, the structural knowledge will aid in the development of enzyme-/family-specific aminopeptidase inhibitors.

## Background

Aminopeptidases (APs) catalyze the sequential removal of amino acids from the unblocked N-termini of protein or peptide substrates, a process necessary for intracellular metabolism [1] and implicated in several human diseases [2]. Most APs are metalloproteases and are classified based on substrate preference towards an acidic, basic or neutral amino acid at the P1 position of the scissile peptide bond. Very few acidic APs are known to date, the most extensively studied being the membrane-bound glutamyl aminopeptidase (ENPEP, also known as aminopeptidase A; EC 3.4.11.7) [3]. ENPEP, a membrane-bound Ca^2+^-activated enzyme, is involved in the renin-angiotensin system (RAS) by catalysing the conversion of angiotensin II to angiotensin III, a key regulator of blood pressure [4,5]. A second, cytosolic acidic AP has been reported in yeast, fungi and mammals, and termed aspartyl aminopeptidase (DNPEP, also known as DAP; EC 3.4.11.21) due to its preference for aspartate over glutamate at the P1 position [6-8]. In mammals, DNPEP is preferentially expressed and has high enzymatic activity in neurons and neuroendocrine tissues [6,9,10]. Its reported conversion of angiotensin I to angiotensin 2–10 [4], and of angiotensin II to angiotensin III [6]*in vitro*, implicates a role in RAS and regulation of blood pressure. Moreover, a mild antagonist effect of DNPEP towards the bone morphogenetic protein signalling pathway has recently been reported [11].

DNPEP is the sole mammalian entry for the M18 metallopeptidase family, which contains ~600 putative members from bacteria and eukaryotes [12]. The M18 family, together with the M20, M28 and M42 families, are classified into the metalloprotease H (MH) clan of proteases on the basis of active site sequence conservation according to the MEROPS database [12,13]. Only a handful of M18 enzymes have been biochemically characterized in any detail; these include yeast vacuole aminopeptidase I (API, also known as Lap4) with a broad substrate specificity for non-polar amino acids [14], as well as yeast yhr113w (also known as Ape4) [7] and mammalian DNPEP which prefer an acidic amino acid. These M18 enzymes are shown to homo-oligomerize, reminiscent of the self-compartmentalization strategy in the well-characterized proteasomes to confer specificity towards unfolded polypeptides and not folded proteins [15]. However, the reported dodecameric form in yeast Lap4 and Ape4 [7,14] contrasts with the proposed octameric form in DNPEP [6].

Little is known about the structure-function relationship of DNPEP and other M18 members, which contain a binuclear metal centre in the active site but lack the signature Zn^2+^-binding sequence motif (HExxH + E) found in other metalloproteases such as ENPEP [16]. Although several conserved histidines essential for catalysis have been identified in human DNPEP [17], their roles are yet to be elucidated. In this study we determined the crystal structure of human DNPEP (hDNPEP) complexed with catalytic Zn^2+^ and substrate analogue L-aspartate-β-hydroxamate (ABH), and confirmed its dodecameric architecture by electron microscopy (EM). The bound ABH ligand highlights the importance of a domain-swapped loop in constructing the active site and provides a structural basis for hDNPEP’s catalytic mechanism and substrate specificity. By comparison with available bacterial M18 structures we further develop a family-wide description of this unannotated peptidase family and suggest unifying catalytic features across the MH clan.

## Results & discussion

### Overall structure of hDNPEP

**Figure 1:**
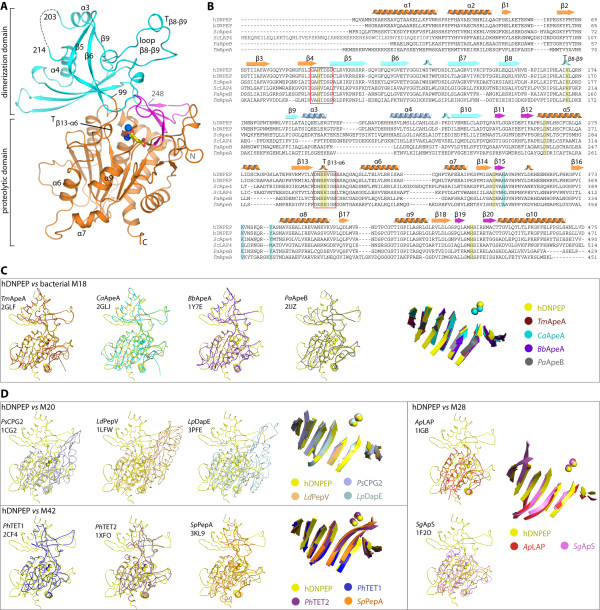
** Overview of hDNPEP structure.** (**A**) hDNPEP protomer organizes into the dimerization (blue) and proteolytic domains, the latter is further comprised of subdomain A (orange) and subdomain B (magenta). Zinc ions are shown as blue spheres and the ABH ligand as yellow sticks. (**B**) Sequence alignment of DNPEP (human, *h*; bovine, *b*) and M18 aminopeptidases from yeast *Saccharomyces cerevisiae* Lap4 (*Sc*LAP4), yeast Ape4 (*Sc*Ape4) and two bacterial enzymes (*Pseudomonas aeruginosa Pa*ApeB and *Thermotoga maritima Tm*ApeA). Secondary structure elements, catalytic residues (yellow) and residues in the P1 substrate pocket (cyan) of hDNPEP are highlighted. Structural superimposition of hDNPEP with bacterial M18 APs (**C**) and with M20, M28 and M42 representatives from the MH clan (**D**) reveals highly conserved topology of the proteolytic domain. The superimposed structures include M18 APs: *Thermotoga maritima* ApeA (*Tm*ApeA), *Clostridium acetobutylicum* ApeA (*Ca*ApeA), *Borrelia burgdorferi* ApeA (*Bb*ApeA), *Pseudomonas aeruginosa* ApeB (*Pa*ApeB); M20 APs: *Pseudomonas* CPG2 (PsCPG2), *Lactobacillus delbrueckii* PepV (LdPepV), *Legionella pneumophila* DapE (*Lp*DapE); M28; *Aeromonas proteolytica* LAP (*Ap*LAP), *Streptomyces griseus* Ap (*Sg*ApS); and M42: *Pyrococcus horikoshii* TET1 and TET2 (*Ph*TET1, *Ph*TET2), *Streptococcus pneumonia* PepA (*Sp*PepA). PDB IDs of all structures are given.

The structure of the hDNPEP·Zn^2+^·ABH complex (Figure 1A), determined at 2.2 Å resolution, is homologous to four unpublished bacterial M18 homologues with undefined enzyme and substrate properties (DALI Z-scores ~40, rmsd 1.9-2.7 Å and sequence identity 23-35%). Superposition of the structures reveals a common two-domain architecture consisting of the proteolytic and dimerization domains (Figure 1A and C), with the active site located in a concave groove at the domain interface. The globular proteolytic domain (aa 7–98 and 249–468 in hDNPEP) features a core nine-stranded β-sheet sandwiched between several α-helices and has a small five-stranded β-subdomain resting on top (Figure 1A). This proteolytic domain is highly similar among all M18 structures (rmsd ~1.5 Å). The dimerization domain, contributed from the central polypeptide stretch (aa 99–248 in hDNPEP), sits on top of the proteolytic domain (Figure 1A). This butterfly-shaped domain is built of two orthogonal β-sheets (five- and three-stranded respectively) that share in common two tilted strands β_5_ and β_6_, and also includes an extended β_8_-β_9_ loop that is important for active site formation (see next sections). Variations in the dimerization domain are observed among M18 enzymes, particularly with the location and spatial orientation of helices α3 and α4 and the connecting loop α3-α4. In hDNPEP loop α3-α4 is longer than the bacterial equivalents (Figure 1B), although it is partially disordered in our structure.

Structural comparison of M18 hDNPEP with members of other MH clan families (M20, M28 and M42) (Figure 1D) shows that the proteolytic domains of all four families can be superimposed well (pairwise rmsd ~2.3 Å), particularly in the core β-sheet and the binuclear metal centre. This structural homology suggests an evolutionarily-conserved strategy for metal coordination and metal-assisted catalysis [13]. Away from the proteolytic domain, however, the four families diverge structurally in the dimerization domain, with M28 members lacking this domain altogether (Figure 1D, right), a fact that is reflected in their different oligomeric states. The hDNPEP dimerization domain exhibits closer topology and orientation to the dodecameric M42 enzymes (Figure 1D, bottom) [18-20], but has distinct fold and tertiary arrangements compared to the counterpart domain in M20 members (Figure 1D, top) that are known monomers or dimers [21,22]. This observation suggests a closer structural relationship of M18 with M42 enzymes, than with M20 or M28 members, a feature not apparent from sequence-based comparisons. This is further supported by M18 and M42 members sharing similar oligomeric assembly and active site architecture (see following sections).

### hDNPEP dodecameric tetrahedron

**Figure 2:**
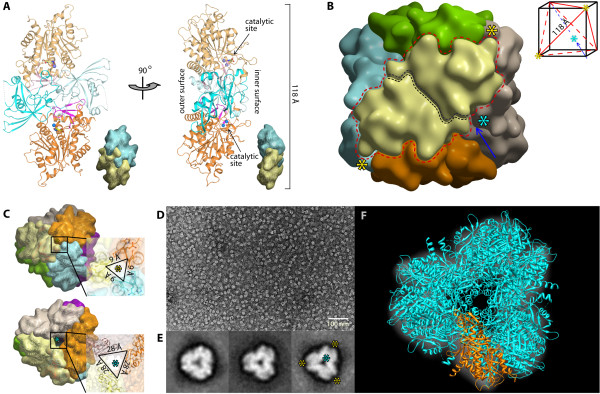
** Dodecameric assembly.** (**A**) A dimer building block of hDNPEP. (**B**) Surface representation of the arrangement of six dimers into a tetrahedron. The dimer building block, each coloured differently, is delineated by a red dotted line. The black-dotted line indicates the monomer-monomer interface within a dimer. Each dimer sits diagonally on six faces of a cubic box that encases the tetrahedron (red line, inset). Asterisks indicate positions of the narrow (yellow) and wide (blue) channels, which are located at 3-fold axes (arrow). (**C**) The openings of the narrow (top) and wide (bottom) channels. (**D**) Electron micrograph of negatively stained hDNPEP. (**E**) Examples of 2D classification images with a view down the wide channel, the 3-fold symmetry imposed in the right panel. (**F**) Fitting of hDNPEP crystal structure onto the 2D projection.

Application of the crystallographic 432 symmetry to the hDNPEP monomer results in a tetrahedron-shaped dodecamer built from six homodimers, a quaternary arrangement similar to M42 enzymes [15,18,19]. Each dimer, with internal two-fold symmetry on both vertical and horizontal axes (Figure 2A), is formed by extensive contacts that involve the swapping of loop β8-β9 between the two subunits (Additional file1, Figure S1). Mediated by four-fold symmetry, the six dimers assemble into a tetrahedron (Figure 2B), with each dimer constituting one edge (~118 Å) of the tetrahedron (Figure 2B, inset). The tetrahedron has a 50 Å-diameter internal cavity harbouring all twelve active sites, which is accessible to the exterior through four wide and four narrow channels situated on the three-fold axes. The entrances of the wide channels, a triangular pore of 28 Å per side, are located at the centre of the four tetrahedron facets (Figure 2B and C, blue asterisks), while the four narrow channels have their openings (9 Å per side) on the four tetrahedron vertexes (Figure 2B and C, yellow asterisks). 

The hDNPEP dodecamer contrasts with an octameric arrangement previously deduced from native PAGE analysis [6]. As an independent verification we performed EM image analysis, revealing one homogeneous particle population on micrographs (Figure 2D) with characteristic patches of density surrounding a hole in the middle, corresponding to the 3-fold symmetrical view down the wide channels at a facet of the tetrahedron complex on the 2D classification (Figure 2E). The tetrahedron shape and dimensions from the EM projection are in excellent agreement with the crystallographic dodecamer (Figure 2F), lending support to its physiologically relevance. While the oligomeric state of the bacterial M18 homologues is not reported, their crystal structures suggest the formation of dodecameric tetrahedrons similar to hDNPEP (Additional file1, Figure S2), pointing towards a common self-compartmentalization strategy for catalysis.

### Architecture of wide and narrow channels

**Figure 3:**
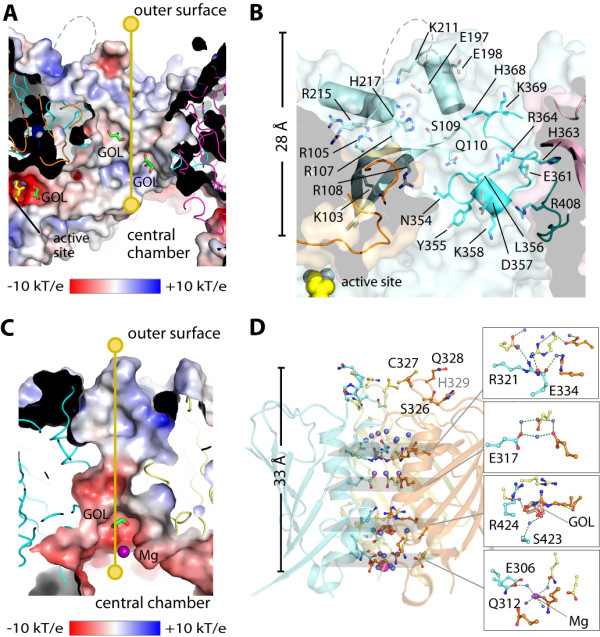
** Architecture of wide (*top*) and narrow (*bottom*) channels.** (**A**) Electrostatic surface of the wide channel with yellow line indicating the 28-Å route connecting the exterior and the central chamber. (**B**) Details of residues lining the wide channel, showing only one set of residues from one dimer. (**C**) Electrostatic surface along the 33-Å length of the narrow channel. (**D**) Details of residues lining the narrow channel. Bound glycerol (GOL) and magnesium (Mg) molecules are shown in stick and sphere, respectively.

We next performed an analysis of the wide and narrow channels in hDNPEP that represent the only access route between the twelve active sites in the central chamber and the exterior. Both channels in M18 hDNPEP are remarkably similar in topology to the M42 dodecameric tetrahedrons. The wide channels, each formed from three dimers (Additional file1, Figure S3), are 20 Å in width and 28 Å in length with a large concave surface at the entrance lined by positively-charged residues (Figures 3A and 3B). This wide channel, supported by the positive electrostatic environment that would complement the substrate acidic N-termini, likely functions as an entrance for unfolded peptide substrates. The transit function, as well as the electrostatic complementarity as a basis for substrate discrimination, has been proposed for M42 tetrahedron aminopeptidases [18,23,24]. Consistent with this theory, mutation of His363 (one of the residues lining the channel) to a non-polar residue has an adverse effect on the hDNPEP kinetic property [17]. 

The narrow channels (Figure 3C) are located at the interface of three monomers that are constituents of different dimers, giving rise to an inner helical bundle with a β-barrel-like outer casing (Figure 3D and Additional file1, Figure S3). The essential nature of this channel has been demonstrated for some tetrahedron aminopeptidases [23]. In hDNPEP, we observed water, glycerol molecules and a hydrated Mg^2+^ ion within this channel (Figure 3C and D), suggesting a possible route for small molecules such as cleaved amino acids to exit after hydrolysis [23]. The narrow channel may also provide a path for the translocation of metal ions (e.g. catalytic zinc), mediated by layers of charged residues within the channel. However, to achieve either transit function, slight conformational changes may be required to open up the channel pore considering its narrow width (~3 Å)(Figure 3C).

### Metal-dependent active site

**Figure 4:**
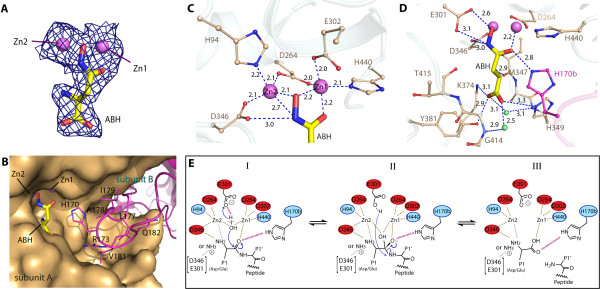
** Active site of hDNPEP.** (**A**) |*F*_*O*_|- |*F*_*c*_| omit map contoured at 3σ for zinc ions and ABH molecules. (**B**) Insertion of the β8-β9 loop from the neighboring subunit (magenta) completes the active site construction. Bonding interactions at (**C**) the binuclear metal catalytic centre and (**D**) the P1 substrate pocket in the hDNPEP structure. (**E**) Proposed catalytic mechanism for hDNPEP. The substrate peptide N-terminus is shown in both amine and its protonated form, which can engage in different interactions.

The active site is defined by the bound substrate analogue ABH and two zinc ions (Zn1 and Zn2) (Figure 4A and B) – the latter likely carried through protein expression and purification, and confirmed by fluorescence absorption profile of the crystals (data not shown). Zn1 and Zn2, bridged by Asp264, are 3.4 Å apart, consistent with the distances observed in other binuclear metalloproteases [13]. Zn1 is further coordinated by Glu302 and His440, and Zn2 by His94 and Asp346 (Figure 4C). These five metal coordinating residues (His94, Asp264, Glu302, Asp346 and His440) form a ‘H.D.E.D.H’ signature strictly conserved among DNPEP paralogues and M18 members (Figure 1B), providing an explanation for the abolished hDNPEP activity by mutations of His94 and His440 [17]. 

Additional coordination to the binuclear zinc is provided by the bound ABH molecule, a competitive inhibitor of hDNPEP [6,17]. ABH binds to the active site with the hydroxamate moiety towards the binuclear metal centre to contribute its carbonyl and hydroxyl oxygen atoms for zinc coordination (Figure 4C), while its amino-acid backbone protrudes into a cavity often known as the P1 substrate pocket (Figure 4B). ABH engages in a number of direct or water-mediated hydrogen bonds to Glu301 and Asp346 via the hydroxamate moiety, and to Tyr381, Lys374 and His349 via the amino acid backbone (Figure 4D). Of particular interest is an interaction between the hydroxamate carbonyl oxygen and His170 from the opposing subunit (His170b) of a dimer (Figure 4D). His170b sits at the tip of the β8-β9 loop from the neighbouring subunit that crosses over to complete the active site (Figure 4B and Additional file1, Figure S1). Such loop swapping to translocate a distant ligand-binding residue into the active site is crucial to hDNPEP catalysis, as evident by a complete abolishment of activity in a His170Phe mutant [17]. This histidine residue is also conserved in M42 enzymes, although in the available M42 structures the equivalent loops are disordered or partially disordered. This disorder could be due to the lack of bound substrate/analogue, suggesting a substrate-induced conformational reorientation is necessary to complete the catalytic centre. Conservation of this histidine therefore implies that the loop-swapped active site is a common structural feature among M18 and M42 dodecamers built from dimeric units.

A possible catalytic mechanism for M18 hDNPEP is proposed (Figure 4E), on the assumption that the hydroxylamine nitrogen and carbonyl oxygen of the ABH hydroxamate represent where the amine and carbonyl groups of the substrate peptide would be coordinated by Zn2 and Zn1, respectively. A nucleophilic water molecule could feasibly occupy the position of the ABH hydroxyl oxygen and would be activated by Glu301 to attack the scissile bond. His170b can function to bind the peptide carbonyl oxygen and stabilize the tetrahedral intermediate. This mechanism is consistent with that proposed for other metallopeptidases [13].

### Structure basis for hDNPEP substrate specificity

The bound ABH provides a template to build dipeptide models of Asp-Ala and Glu-Ala into the active site in order to rationalize hDNPEP substrate specificity. For both peptides, the Asp and Glu sidechains fit into the P1 substrate pocket without steric constraints, while the mainchain is modeled onto the hydroxamate group of ABH in a position optimal for hydrolysis. The P1 substrate pocket (Additional file1, Figure S4A) is created by strand β15 and the β16-α12 and β17-α13 loops, with the β17-α13 loop lining the wall and restricting the dimensions of the pocket. This limited space disfavours bulky hydrophobic residues, as illustrated by a structural comparison with the P1 pockets in M28 neutral aminopeptidases where the equivalent loop is displaced away from the P1 pocket thereby generating a large cavity for bulky residues such as Phe and Met (Additional file1, Figure S4B and C).

**Figure 5:**
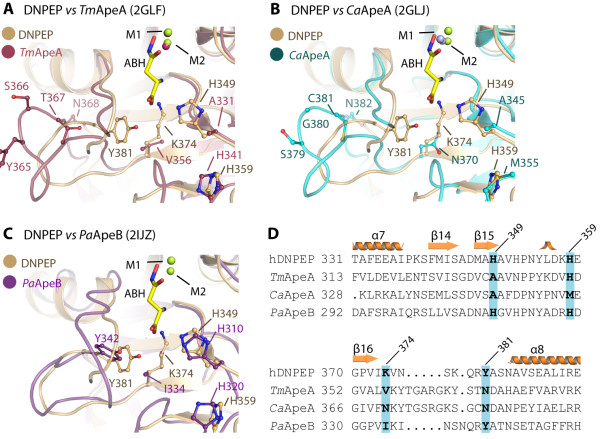
** Structural comparison of the P1 pockets in hDNPEP and bacterial M18 members.** (**A**-**C**) Three bacterial M18 AP structures (PDB ids in brackets) are superimposed onto hDNPEP, with particular focus on the P1 substrate pocket. This highlights variations not only in shape but also residue compositions for the P1 pocket, and may be correlated with different substrate specificities and enzyme activities among M18 enzymes. (**D**) A structure-based sequence alignment shows no conservation of four key residues of the hDNPEP P1 pocket among the bacterial M18 enzymes, in particular Lys374 likely to be a determinant for acidic amino acid preference.

The modelled Asp and Glu sidechains can engage in slightly different interactions with hDNPEP (Additional file1, Figure S5). While the Asp carboxylate feasibly interacts with Lys374 and forms water-mediated hydrogen bonds with His349, the longer Glu sidechain can further penetrate this cavity and interact directly with Lys374, His349 and the nearby Tyr381. Our substrate models suggest that the strict preference for an acidic amino acid at the P1 position is conferred by positively-charged and polar residues, such as Lys374 and His349. The use of electrostatic complementarity for substrate selectivity has precedence in the M42 peptidase *Sp*PepA [20]. Consistent with this strategy, mutation of His349 in hDNPEP has been shown to weaken substrate binding affinity [17]. Furthermore, the conservation of Lys374 only in M18 members with acidic aminopeptidase activity (e.g. yeast Ape4), but not in M18 ‘promiscuous’ peptidases (e.g. yeast Lap4, where the equivalent is Ser) (Figure 1B), provides a structure-based criteria to classify putative M18 sequences into potential aspartyl aminopeptidases (Lys374 conserved) or non-aspartyl aminopeptidases (Lys374 not conserved), facilitating subsequent enzymatic characterization. Using this criteria we propose that the structurally characterized bacterial M18 members, where the equivalent Lys374 positions are substituted (Figure 5), are unlikely to be aspartyl aminopeptidases. 

## Conclusion

In summary, we provide a structural annotation of the M18 metallopeptidase family, highlighting common catalytic residues and oligomeric properties. In particular, a loop-swapped active site utilizing a residue from an adjacent subunit for catalysis is likely a common characteristic among M18 and M42 dodecameric aminopeptidases. Furthermore, the bound substrate analogue in the active site provides insight into the reaction mechanism and substrate specificity for hDNPEP, facilitating the next steps in the development of family-specific small-molecule binders to further probe its cellular role in metabolic pathways and disease.

## Methods

### Cloning, expression and protein purification

A DNA fragment encoding hDNPEP aa 1–468 (Uniprot ID: Q9ULA0) was sub-cloned into the pNIC-CTHF vector, incorporating a C-terminal His_6_-tag and TEV protease site. The recombinant protein was expressed in *E. coli* BL21(DE3)-R3 by induction with 0.1 mM IPTG overnight at 18°C. Cells were harvested and homogenized in lysis buffer (50 mM HEPES pH 7.5, 500 mM NaCl, 5 mM imidazole, 5% glycerol). Protein was purified by affinity (Ni-Sepharose) and size exclusion chromatography (Superdex 200). The affinity tag was removed by His-tagged TEV protease and the TEV-cleaved protein was passed over Ni-Sepharose resin. Purified protein was stored at −80°C in 10 mM HEPES, pH 7.5, 500 mM NaCl, 5% glycerol and 0.5 mM TCEP.

### Crystallization and data collection

hDNPEP (10 mg/ml) was pre-incubated with 5 mM L-aspartate-β-hydroxamate (ABH) and crystallized by sitting drop vapour diffusion at 20°C in a 150-nl drop by mixing protein and reservoir solution (15% *w/v* PEG 3350, 0.25 M MgCl_2_ and 0.1 M Tris–HCl pH 8.0) in a 2:1 ratio. Crystals were cryo-protected with mother liquor supplemented with 25% glycerol and flash-cooled in liquid nitrogen. Diffraction data were collected at Diamond Light Source beamline I03, and processed and scaled with MOSFLM and SCALA from the CCP4 suite [25].

### Structure determination

hDNPEP crystals belong to the *F*-centered cubic spacegroup *F*432 with unit cell parameters a,b,c = 224.6 Å and α,β,γ = 90.0°. The asymmetric unit contains one hDNPEP protomer. The structure was solved by molecular replacement using PHASER [26] and the *Pseudomonas aeruginosa* M18 structure (PDB id: 2IJZ) as search model. Density modification was performed using DM [27] and improved phases were used for automated model building with ARP/wARP [28]. The structure was refined using REFMAC [29] and rebuilt with COOT [30]. Residues 1–6 and 204–213 are disordered and not included in the final model. Data collection and refinement statistics are summarized in Table1. 

**Table 1:** Data Collection and Refinement Statistics

	**hDNPEP·Zn**^**2+**^**·ABH**
**PDB accession code**	4DYO
***Data collection***	
Beamline	Diamond Light Source, I03
Wavelength (Å)	0.9763
Spacegroup	*F* 432
Resolution range^a^ (Å)	56.11 – 2.20 (2.32 – 2.20)
Unit cell dimensions	*a* = *b = c* = 224.60 Å; α = β = γ = 90.0°
No. unique reflections^a^	32,192 (4,486)
Completeness^a^ (%)	99.6 (97.5)
I/σI^a^	10.4 (2.2)
R_merge_^a^ (%); R_pim_^a^ (%)	17.9 (83.3); 5.5 (29.7)
Redundancy^a^	10.8 (8.0)
Wilson B factor (Å^2^)	28.5
***Refinement***	
No. atoms in refinement (P/L/M/O)^c^	3585/10/2/358
R_fact_ (%)	15.5
R_free_ (%)	19.5
B_f_ (P/L/M/O)^c^ (Å^2^)	26/30/28/28
rms deviation bond length^b^ (Å)	0.015
rms deviation bond angle^b^ (°)	1.5
***Molprobity***	
Ramachandran favoured	97.1
Ramachandran allowed	99.8

^a^ Values in brackets show the statistics for the highest resolution shells.

^b^ rms, root-mean-square.

^c^ P/L/M/O indicate protein, ligand molecules in the active sites, metal zinc ions and other molecules, respectively.

### Electron microscopy

hDNPEP at ~0.7 μM was applied to EM grids and stained with 2% uranyl acetate. Electron micrographs were recorded (x 45,000) using a FEI-Phillips CM120 EM. Images were digitized on a Nikon Super Coolscan 9000 (step size of 12.5 μm with a pixel size of 2.78 Å). The WEB and SPIDER software [31] were used for image processing. 4,736 particles were windowed, subjected to reference-free alignment, and sorted into classes using the K-means clustering method [32]. Manual fitting of the hDNPEP crystal structure into the 2D map was achieved using CHIMERA [33].

## Footnotes

The atomic coordinates and structure factors have been deposited in the Protein Data Bank (http://www.rcsb.org/) with accession number 4DYO.

## Competing interests

The authors declare that they have no competing interests.

## Authors’ contributions

KLK, UO and WWY designed the experiment. AC, ESP, ADR, FvD, CVB performed the experiment. AC, ADR, WWY analyzed the data. AC and WWY wrote the manuscript. All authors read and approved the final manuscript.

## Supplementary Material

Additional file ** Figure S1.** Domain swapping inthe hDNPEP dimer. **Figure S2.** Tetrahedron complexes of available bacterial M18 structures. **Figure S3.** Architecture of the wide and narrow channels. **Figure S4.** hDNPEP P1 substrate pocket. **Figure S5.** Substrate peptide modelling into hDNPEP [34-37]. Click here for file
