# Supplementary material for: Structure of human aspartyl aminopeptidase complexed with substrate analogue: insight into catalytic mechanism, substrate specificity and M18 peptidase family
Source: BMC Struct Biol. 2012 Jun 21;12:14. doi: 10.1186/1472-6807-12-14 (PMC3472314; doi:10.1186/1472-6807-12-14)
Supplement: Additional file — Figure S1. Domain swapping inthe hDNPEP dimer. Figure S2. Tetrahedron complexes of available bacterial M18 structures. Figure S3. Architecture of the wide and narrow channels. Figure S4. hDNPEP P1 substrate pocket. Figure S5. Substrate peptide modelling into hDNPEP [34-37]. [file 1472-6807-12-14-S1.doc]

# Structure of human aspartyl aminopeptidase complexed with substrate analogue: insight into catalytic mechanism, substrate specificity and M18 peptidase family

**Apirat Chaikuad^1^, Ewa S. Pilka^1^, Antonio De Riso^2^, Frank von Delft^1^, Kathryn L. Kavanagh^1^, Catherine Vénien-Bryan^2^, Udo Oppermann^1,3^ & Wyatt W. Yue^1^**

From ^1^Structural Genomics Consortium, Old Road Research Campus Building, Oxford, UK OX3 7DQ. ^2^Laboratory of Molecular Biophysics, Department of Biochemistry, Oxford, UK OX1 3QU. ^3^Botnar Research Centre, Oxford Biomedical Research Unit, Oxford, UK OX3 7LD

Address correspondence to Wyatt W.Yue, Tel: +44 1865 617757; Fax: +44 1865 617580; Email: [wyatt.yue@sgc.ox.ac.uk](mailto:wyatt.yue@sgc.ox.ac.uk)

**Additional file 1**

*This document comprises the following sections:*

**Figure S1.** Domain swapping inthe hDNPEP dimer.

**Figure S2.** Tetrahedron complexes of available bacterial M18 structures.

**Figure S3.** Architecture of the wide and narrow channels.

**Figure S4.** hDNPEP P1 substrate pocket.

**Figure S5.** Substrate peptide modelling into hDNPEP.

**Supplemental references**


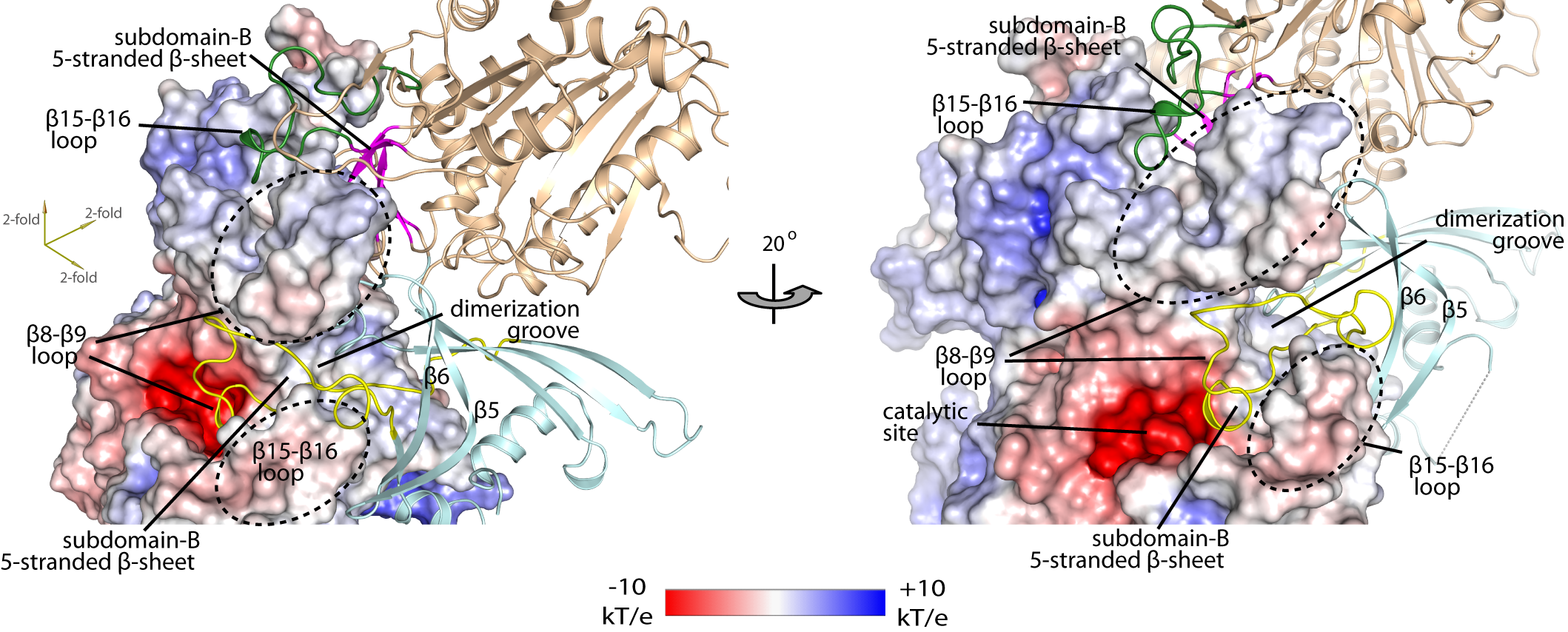


**Figure S1.** Domain swapping in the hDNPEP dimer. The figure shows a region of the dimer interface, with one subunit of the dimer displayed in surface representation and the other in ribbons. Dimer formation depends on the shape complementarity between the two subunits by means of the exchange insertion of loop β8-β9 from the dimerization domain of one subunit, into a groove above subdomain B in the proteolytic domain of the other subunit (and vice versa).


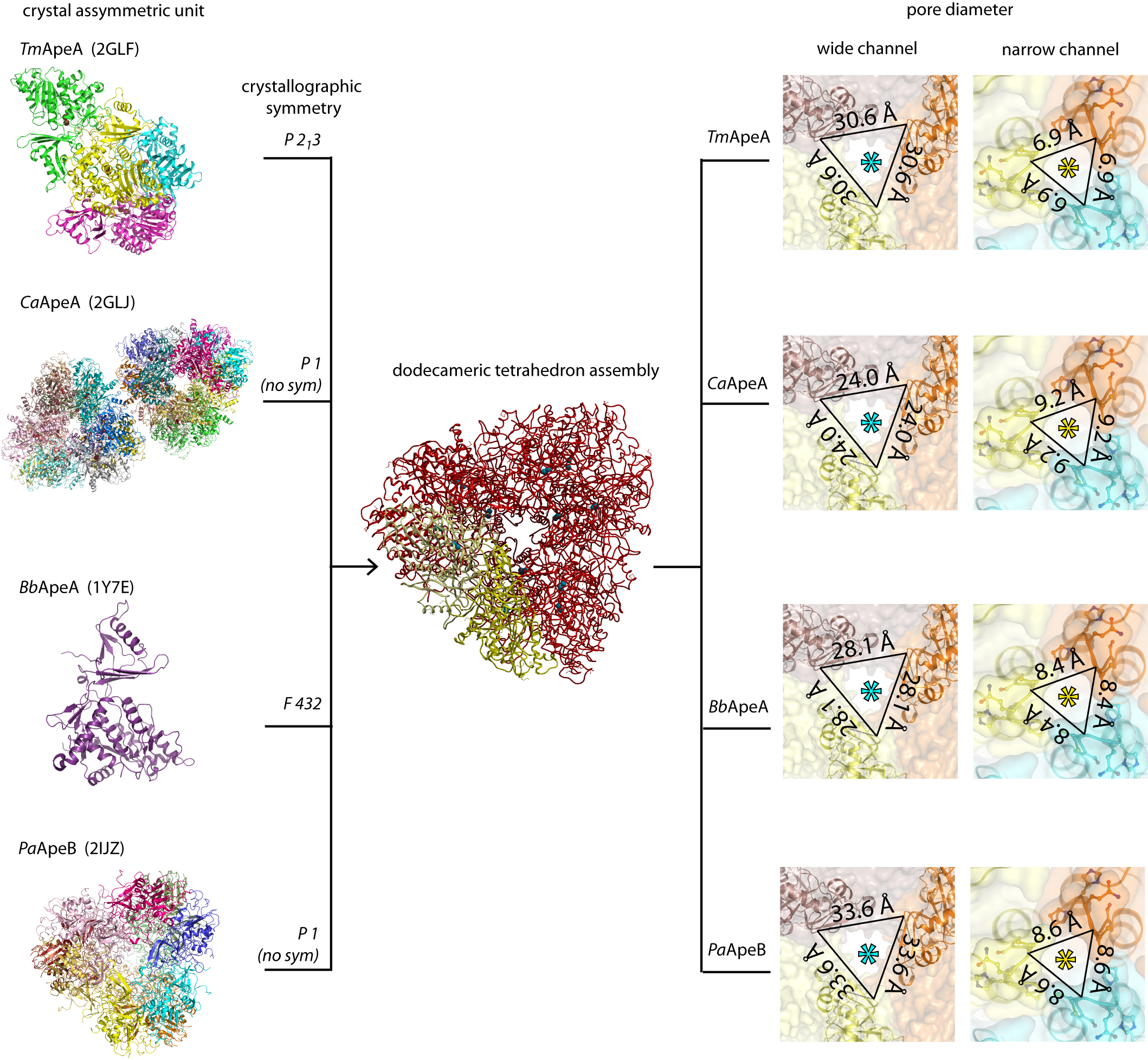


**Figure S2.** Tetrahedron complexes of available bacterial M18 structures. Dodecameric assemblies of these bacterial enzymes, which are either observed in the asymmetric units or built upon an application of crystallographic symmetry, are highly similar to that of hDNPEP. These tetrahedron complexes are constituted from a dimer building block, shown in yellow and pale yellow corresponding to each monomer in the middle panel. On the right panel, approximate diameters of their wide and narrow channels are shown. PDB IDs for each structure are in brackets. *Tm*, *Thermotoga maritima*; *Ca*, *Clostridium acetobutylicum*; *Bb*, *Borrelia burgdorferi*; *Pa*, *Pseudomonas aeruginosa*.


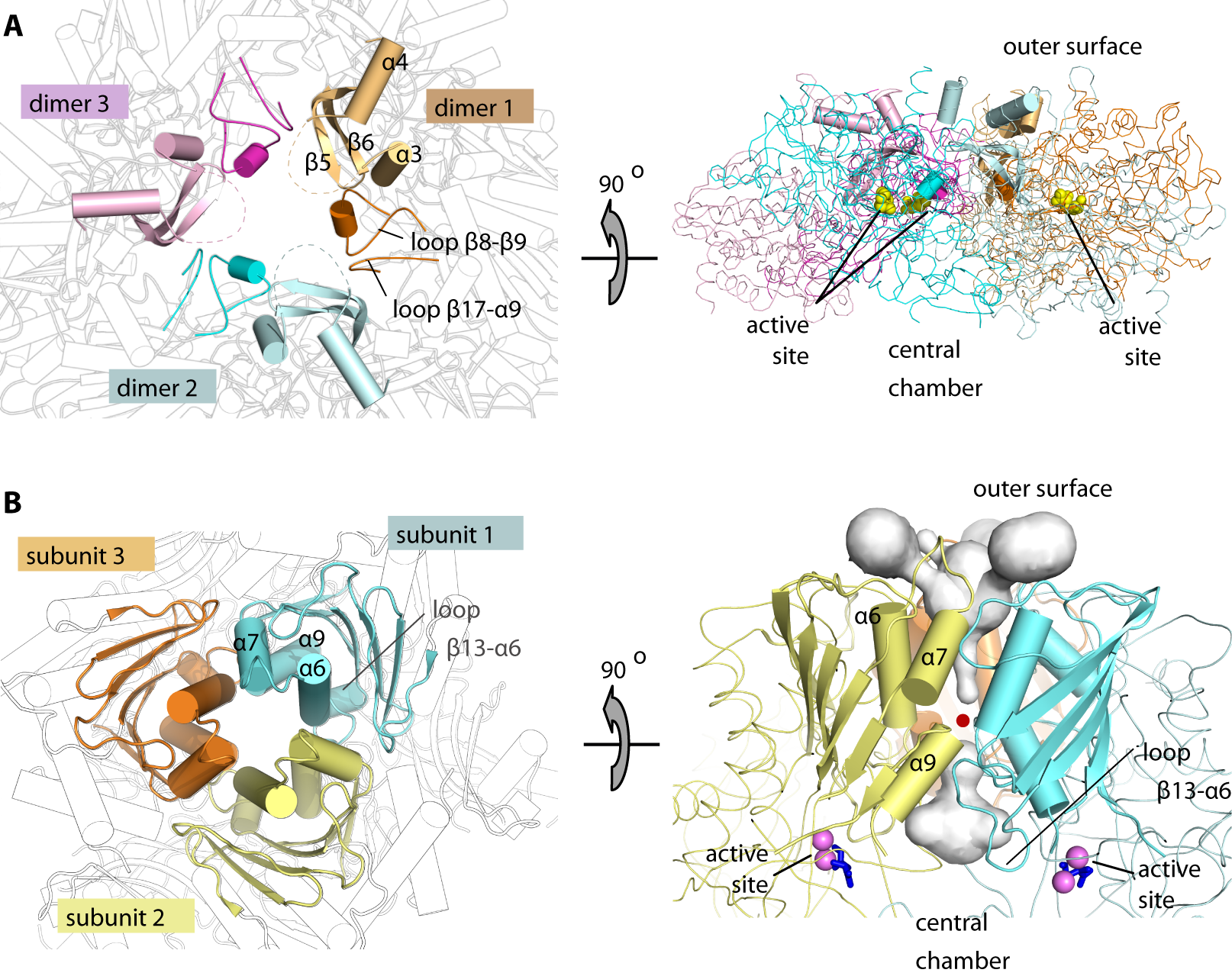


**Figure S3.** Architecture of the wide and narrow channels. *A*, The wide channel is located in between three dimers, and constructed mainly from the β_15_-β_16_ and β_17_- α_9_ loops of one subunit and the β_5_, β_6_ and α_3_ of the other subunit from each dimer. It has a large concave surface at the outer opening pore connected to a shallow portal that acts as a barrier to the central chamber and three active sites, which are located on a perimeter at ~30 Å away from the centre. *B*, The narrow channel adopts the β-barrel like architecture, which is built from three subunits from three different dimers. Each contributes a helical bundle (α6, α 7 and α9) forming an inner core surrounded by the main β-sheets of the proteolytic domain. A possible delivery route between the exterior and the central chamber is predicted by CAVER [34], as shown by white surface. The calculation is performed at the narrowest point (red dot) with ~3-Å width.


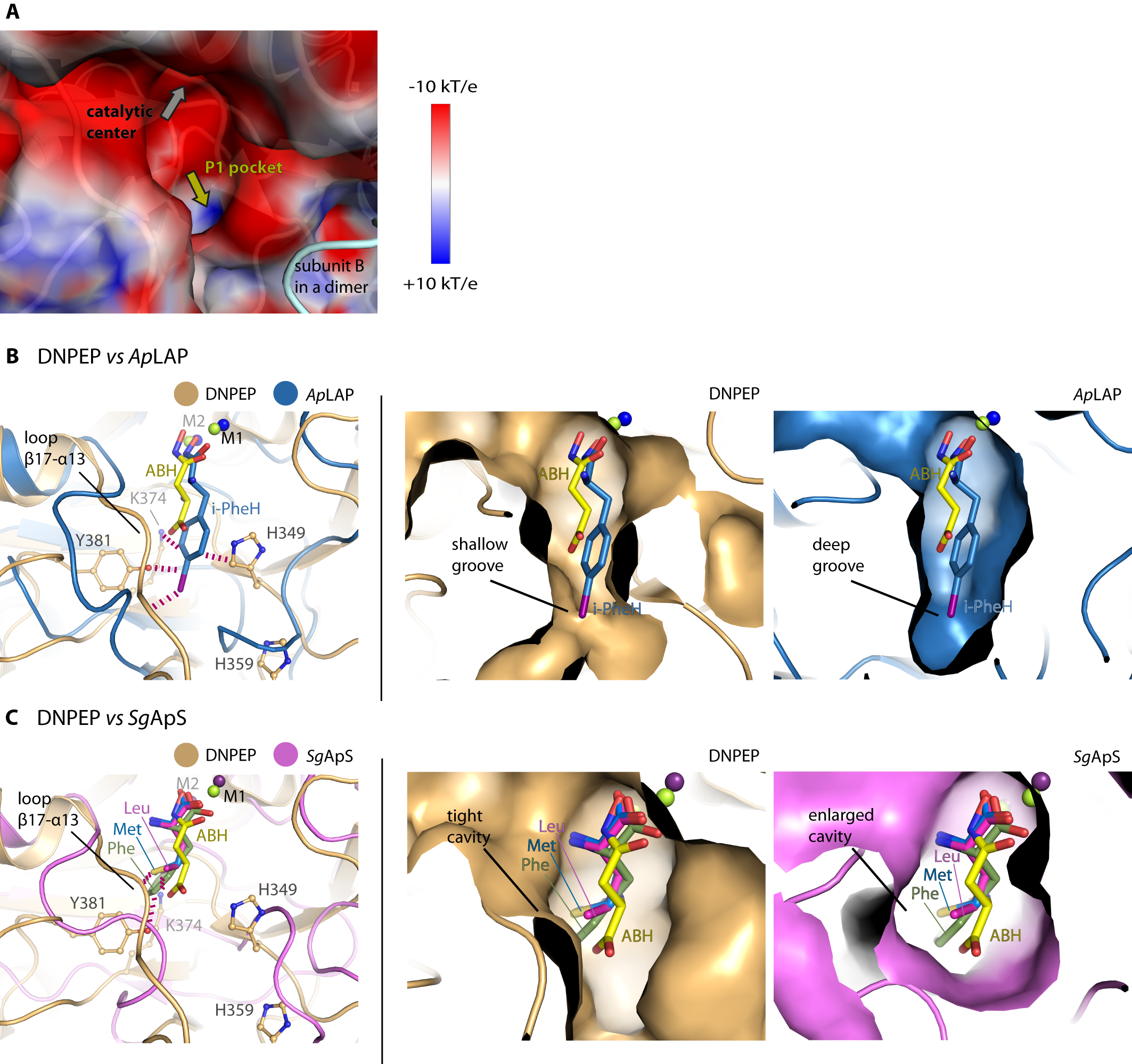


**Figure S4.** hDNPEP P1 substrate pocket. *A*, Positively-charged characteristics of hDNPEP P1 pocket that complement the acidic nature of aspartate/ glutamate sidechains. *B and C*, Superimposition of hDNPEP-Zn^2+^-ABH complex with structures of bacterial M28 APs in complexes with either substrate analogue (iodo-phenylalanine hydroxamate; i-PheH) or amino acid products [35-37] reveals significant structural variability in the P1 pocket region, especially with regard to the hDNPEP β17-α13 loop. This region in M28 enzymes, which accepts a broad range of nonpolar amino acid, is rather extended, and hence creates space for large hydrophobic amino acid. This is in contrast to the rather tight and shallow P1 substrate groove of hDNPEP that leads to possible steric clashes with bulky amino acid substrates.


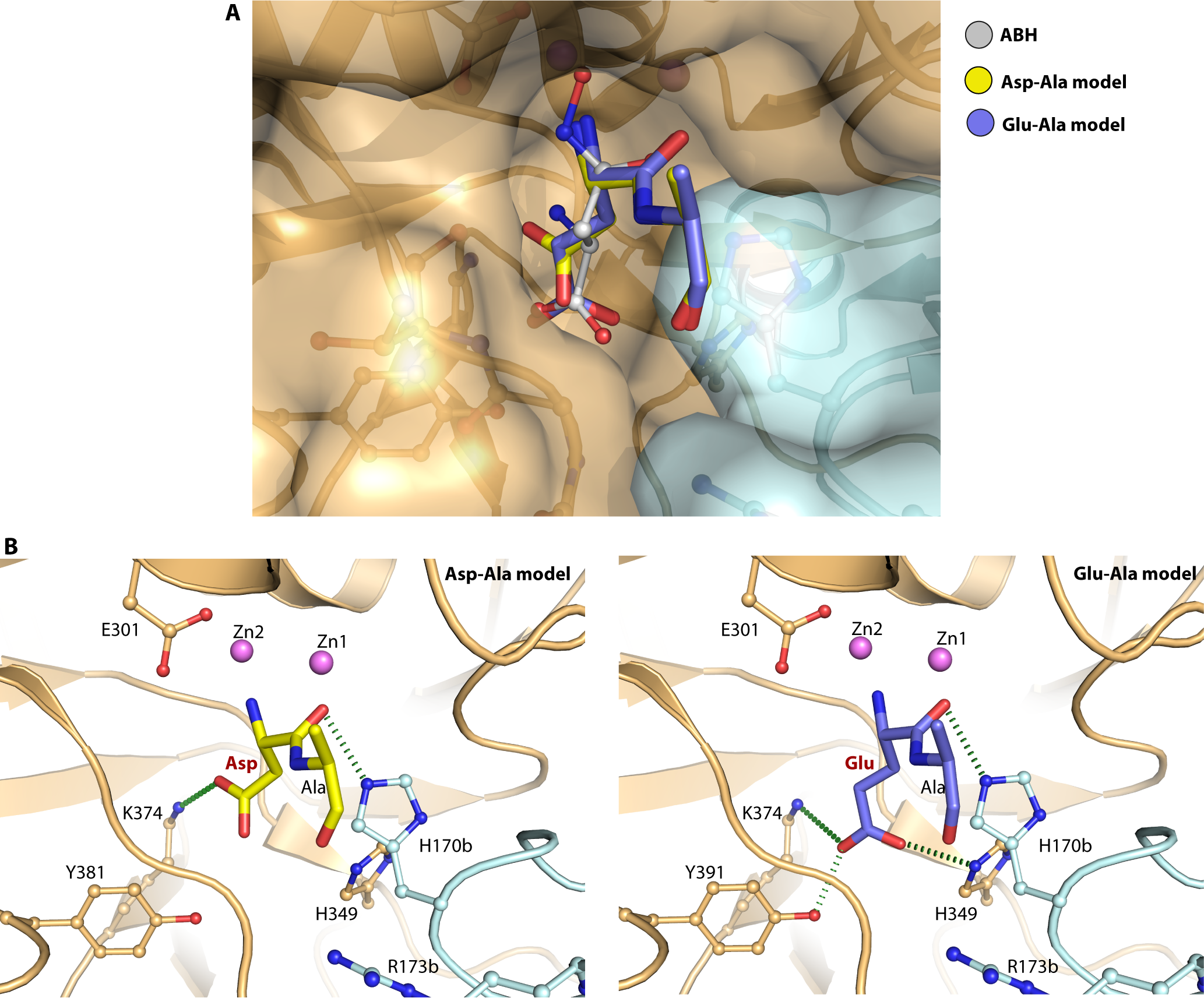


**Figure S5.** Substrate peptide modelling into hDNPEP. *A,* Two dipeptides, Asp-Ala and Glu-Ala, are modeled into hDNPEP active site based on the bound ABH ligand. *B*, Possible direct hydrogen bonds between the aspartate and glutamate side chains with residues in the P1 pocket, including Lys374, Tyr381 and His349, are shown. Note a common interaction between Lys374 and both modelled P1 amino acids. The peptide mainchain carbonyl is located in proximity to His170b, which is required for catalysis.

**Supplemental references**

34. Petrek, M., Otyepka, M., Banas, P., Kosinova, P., Koca, J., and Damborsky, J. (2006) *BMC Bioinformatics* **7**, 316

35. Gilboa, R., Greenblatt, H. M., Perach, M., Spungin-Bialik, A., Lessel, U., Wohlfahrt, G., Schomburg, D., Blumberg, S., and Shoham, G. (2000) *Acta crystallographica* **56**(Pt 5), 551-558

36. Gilboa, R., Spungin-Bialik, A., Wohlfahrt, G., Schomburg, D., Blumberg, S., and Shoham, G. (2001) *Proteins* **44**(4), 490-504

37. Chevrier, B., D'Orchymont, H., Schalk, C., Tarnus, C., and Moras, D. (1996) *Eur J Biochem* **237**(2), 393-398
